# Carrion Increases Landscape‐Scale Scavenger Activity and Interactions

**DOI:** 10.1002/ece3.72059

**Published:** 2025-08-23

**Authors:** Patrick B. Finnerty, Niraj Y. Meisuria, Luke J. Baker, Rhys J. Cairncross, Emma Spencer, Mathew S. Crowther, Thomas M. Newsome

**Affiliations:** ^1^ School of Life and Environmental Sciences The University of Sydney Sydney New South Wales Australia

**Keywords:** carrion availability, competitive interactions, road ecology, scavenger activity, temporal partitioning

## Abstract

Carrion is a nutrient‐rich but spatiotemporally unpredictable resource that supports diverse trophic interactions, and its consumption plays a key role in energy recycling within ecosystems. Although previous research has almost exclusively examined scavenger activity at carcasses, the broader impacts of fluctuating carrion availability on landscape‐scale activity patterns and scavenger competitive interactions remain largely unexplored. Here, we investigate whether carrion provisioning influences broader road use activity in two competing facultative scavenger species: dingoes ( *Canis dingo* ), an apex scavenger, and red foxes ( *Vulpes vulpes* ), a mesoscavenger. Using camera traps, we monitored the activity of the two focal species on roads before and after the placement of 20 experimentally deployed eastern grey kangaroo ( *Macropus giganteus* ) carcasses in the surrounding landscape. Carcasses—also monitored using camera traps—were positioned at least 1 km apart at similar distances from roads across open forest and grassland habitats, in summer and winter in southeast Australia. Both dingoes and red foxes exhibited increased road use following carcass deployment, with red fox responses varying seasonally—showing a greater increase in road use during winter months. Overlap in activity times between the focal species on roads also increased post‐carcass deployment—especially during the winter month; however, peak activity times remained slightly offset. The focal species temporal overlap at carcasses was also greater in the winter season. Our study provides novel insights into the ecological significance of carrion beyond local carcass sites, highlighting its potential role in influencing broader scavenger activity patterns. We also reveal that peak road use may serve as a reliable proxy for peak scavenging times in systems where carcasses are available near roads, given the overlap in diel activity patterns of both dingoes and red foxes on roads and at carcasses. These findings emphasize the need for further research to examine how carrion availability shapes scavenger dynamics at landscape scales.

## Graphical abstract

This study explores how carrion availability drives landscape‐scale activity and interactions between two facultative scavengers: dingoes and red foxes. Experimental carcass provisioning across two seasons in southeast Australia revealed increased road use and temporal overlap between species, particularly in cooler months. These findings demonstrate that carrion shapes not just local scavenging behavior, but broader landscape‐scale activity and interspecific dynamics.

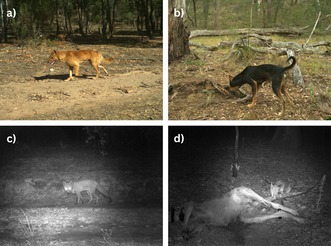

## 1 Introduction

Carrion is a ubiquitous resource across almost all ecosystems globally, entering landscapes via natural animal deaths, vehicular collisions (i.e., roadkill), lethal pest control (Englefield et al. 2018), and a variety of other anthropogenic threats such as collisions with infrastructure, electrocutions, and poisoning events. As a nutrient‐dense resource, carrion supports a variety of trophic groups within complex food webs (Mateo‐Tomás et al. 2015; Wenting et al. 2024). Moreover, carrion consumption presents a crucial role in energy recycling within ecosystems (Barton et al. 2013; DeVault et al. 2004). As a foraging resource, however, carrion availability is often highly variable, fluctuating unpredictably across both time and space (Moleón et al. 2019). It remains unclear whether scavengers adjust their movements and activity patterns to actively locate carrion or if they primarily encounter it opportunistically while focusing on other food resources. Unraveling these dynamics could demonstrate behavioral plasticity among scavengers. Additionally, understanding how fluctuating carrion presence may influence scavenger activity rates could offer deeper insights into the temporal overlaps and competitive interactions that shape the complex ecological relationships among scavenging guilds.

To infer species activity rates within a given landscape, the use of linear features such as roads—known to facilitate movement across various species, including predators and facultative scavengers—has become a common approach (Dickie et al. 2017; Hill et al. 2021; Johnson‐Bice et al. 2023; Petridou et al. 2023; Zimmermann et al. 2014). Roads may serve as corridors for movement but also as indicators of foraging behavior, with scavenger road use potentially increasing in response to heightened carrion availability (Baruzzi et al. 2022). Given that road use has previously been used as a proxy for predator foraging activity (Petridou et al. 2023; Raiter et al. 2018), tracking scavengers along roads may provide valuable insights into broader‐scale shifts in scavenging dynamics across a landscape. Additionally, as roads are known hotspots of wildlife mortality (Moore et al. 2023), they can often contain higher carrion loads than surrounding habitats, further supporting their use in assessing scavenger activity (e.g., roadkill studies).

In many systems globally, both apex and mesoscavengers exploit carrion resources. These groups can both contribute to carrion consumption, but their responses can differ. Apex scavengers often monopolize carcasses and may alter their spatial and temporal activity patterns to track carrion pulses, whereas mesoscavengers may adjust their activity to avoid dominant competitors or exploit carrion in their absence (Finnerty et al. 2024). For example, grey wolves ( *Canis lupus* ) in North America modify their movements to locate and monopolize large ungulate carcasses, particularly in winter (Wilmers et al. 2003), while mesoscavengers such as red foxes ( *Vulpes vulpes* ) and coyotes ( *Canis latrans* ) frequently delay carcass visits to avoid direct interactions with wolves (Klauder et al. 2021). Similarly, in southern Africa, spotted hyenas ( *Crocuta Crocuta* ) often arrive at carcasses soon after death to monopolize resources, whereas black‐backed jackals (*Lupulella mesomelas*) adjust their activity to scavenge opportunistically when these dominant scavengers are absent (Jones et al. 2015; Melville et al. 2023). Therefore, understanding how apex and mesoscavengers differ in their changes in road use and in the timing of carcass visitation in response to carrion availability can provide insights into how resource pulses shape their movement ecology, competitive interactions, and broader ecosystem processes.

Within an Australian context, here we focus on the scavenging rates and activity patterns of dingoes ( *Canis dingo* ), a common native apex scavenger (Spencer and Newsome 2021) and invasive red foxes, a common mesoscavenger (Fleming et al. 2021; Vandersteen et al. 2023). Dingoes and red foxes are both nocturnal (Díaz‐Ruiz et al. 2016; Meek et al. 2024; Newsome et al. 2024; Spencer and Newsome 2021) and while dietary overlap between the two species is variable and depends on prey availability, studies have shown that both often target similar prey items such as small to medium‐sized mammals, leading to dietary competition where dingoes can suppress red fox foraging success and influence their prey choices (Davis et al. 2015; Mitchell and Banks 2005). Such competition, combined with dingo top‐down regulation of red foxes, may lead to temporal partitioning of resources between the two species. Fluctuating foraging resources, such as carrion pulses, may influence the strength of this top‐down control, with increased carrion availability either reinforcing dingo suppression of red fox activity or providing alternative foraging opportunities that reduce direct interactions (Greenville et al. 2014). Although temporal partitioning has been inferred from camera trap studies on roads (Gabriele‐Rivet et al. 2020; Wagnon and Serfass 2017) and separately on carcasses (Forsyth et al. 2014), no study to date has simultaneously monitored their use of both roads and carcasses. This limits our ability to understand how they might respond to the presence of carrion in the landscape.

In monitoring dingo and red fox scavenging activity on experimentally placed carcasses, alongside their nearby road use activity before and after carcass placement, we aimed to uncover how they utilize these resources and whether there are any shifts in activity times associated with carcass availability. Given seasonality is well documented to affect dingo and red fox scavenging rates (Needham et al. 2014; Spencer and Newsome 2021; Vandersteen et al. 2023), we also compared scavenging and road use activity across both summer and winter seasons. Based on current knowledge of dingo and red fox activity and scavenging patterns, we tested the following predictions:

- *Increased road activity following carcass deployment*: as carrion represents a high value but ephemeral resource, its provisioning is expected to alter predator movement patterns. We predicted that both dingoes and red foxes would increase their road use activity following carcass deployment compared to pre‐deployment periods, reflecting behavioral plasticity in response to novel foraging opportunities and consistent with optimal foraging theory (Charnov 1976), which predicts increased activity in areas of elevated resource availability.
- *Temporal partitioning on roads*: due to intraguild competition and risk avoidance, we predicted that dingoes and red foxes will exhibit temporal segregation in their road use under baseline conditions (pre‐carcass deployment), with activity peaks offset to reduce direct encounters (consistent with previous findings; Greenville et al. 2014). However, following carcass deployment, we expect this temporal separation to diminish, as increased carrion availability reduces competitive pressure and promotes temporal overlap due to shared attraction to the resource.
- *Temporal partitioning at carcass sites*: similarly, peak carrion usage times will differ between dingoes and red foxes, consistent with temporal partitioning common between dingoes and red foxes (Greenville et al. 2014), including at carrion (Forsyth et al. 2014). However, some temporal overlap is expected given multiple carcasses available within a landscape may allow dingoes and red foxes to access different carcasses at the same time; a low density of dingoes may also limit their ability to monopolize all carcass resources.

## 2 Methods and Materials

### 2.1 Study Area

**FIGURE 1:**
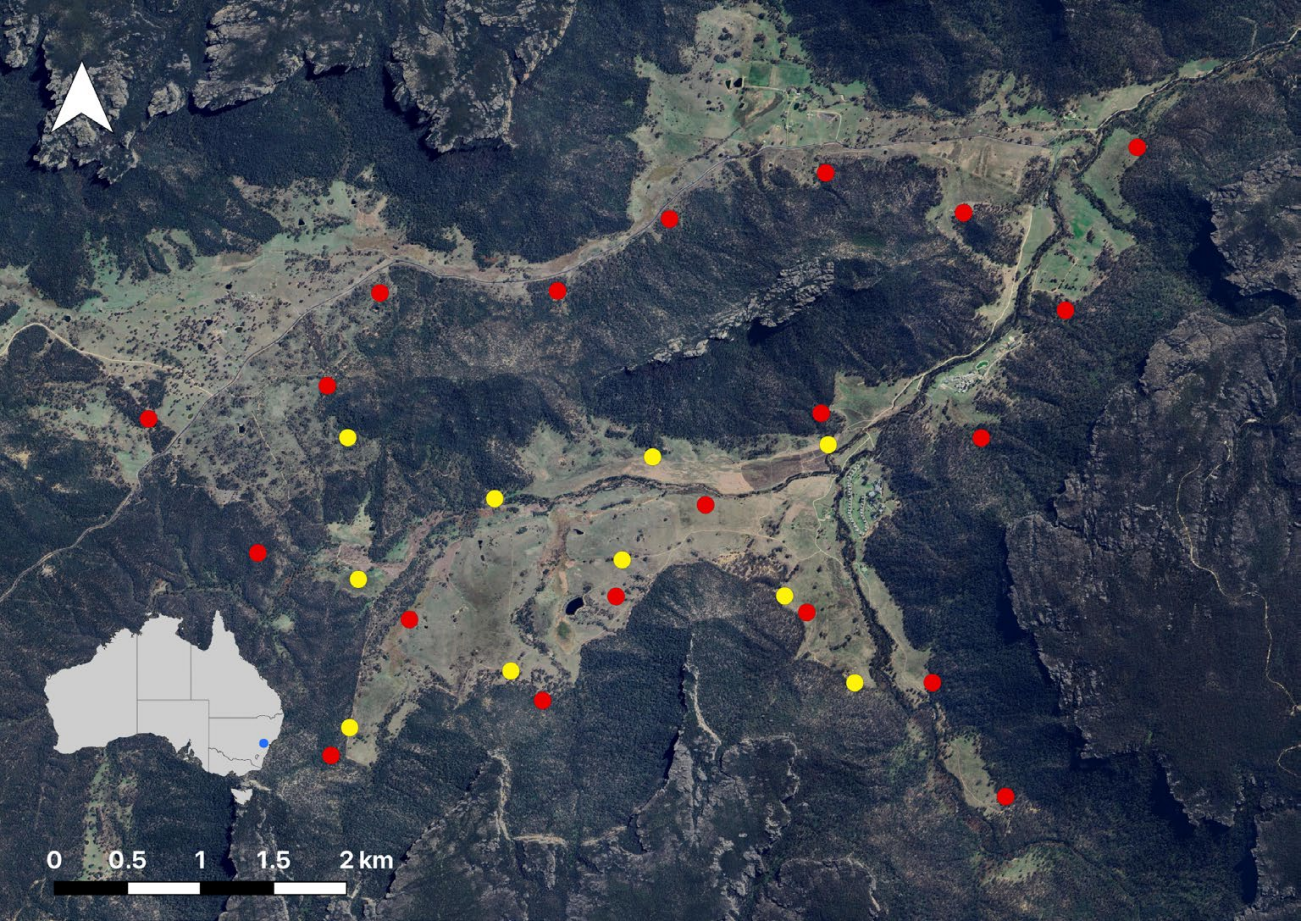
Location of the study area in the Wolgan Valley, Blue Mountains in southeastern NSW, Australia, and an example of the specific carcass monitoring locations (red) and road monitoring locations (yellow) in a single season. Monitoring was conducted over two 30‐day periods, across a summer (23 January—22 February 2019) and a winter (11 July—10 August 2019) season. In each season, 20 adult eastern grey kangaroo carcasses were deployed. Figure generated using QGIS 3.34.3‐Prizren.

This study was conducted in the Wolgan Valley, located on the border of the Greater Blue Mountains National Park, southeast New South Wales (Figure 1). The study area spans approximately 50 km^2^ at an elevation of 540–680 m. The climate is temperate, with highest mean temperatures in January and July of 27°C and 11°C respectively. Average temperatures in the winter season range from 1.0°C to 12.5°C. Average temperatures in the summer season range from 13.0°C to 26.4°C. A diverse mix of habitats occurs within the landscape, including agricultural land consisting of pastures within a matrix of native grassland, open woodland, and forest habitats containing both exotic (e.g., *Microlaena stipoides* ) and native grasses (e.g., *Austrodanthonia* sp. and *Themeda triandra* ) and a variety of *Eucalyptus* species (e.g., *Eucalyptus viminalis* and *E. haemastoma* ).

### 2.2 Ethics and Permits

Ethics approvals (Project number: 2017/1173) and scientific licence and collection permits (SL 101901, New South Wales Office of Environment and Heritage) were obtained to undertake the fieldwork. Carcasses of eastern grey kangaroos ( *Macropus giganteus* ) were sourced fresh and locally from legally approved shooting operations that are conducted to control or harvest overabundant kangaroo populations.

### 2.3 Road and Carcass Monitoring

Road monitoring was conducted 30 days prior and 30 days post‐carcass deployment in a summer (24 December 2018–22 February 2019) and winter (11 June 2019–10 August 2019) season using 10 non‐baited camera traps deployed along dirt roads that traversed the study area (Figure 1). The dirt roads monitored have very low traffic, with typically fewer than 10 vehicles per day. Each road camera was positioned at least 1 km from the next, consistent with similar Australian predator–prey road use surveys (Greenville et al. 2014). This spacing was also deemed appropriate to capture dingo and red fox responses to carcasses within the 50 km^2^ study area.

Carcass monitoring was conducted over two 30‐day periods, in a summer (23 January—22 February 2019) and winter (11 July—10 August 2019) season. In each season, 20 adult eastern grey kangaroo carcasses were evenly distributed across a matrix of grassland and open forest habitats. Camera traps were deployed on each carcass (*n* = 20). Carcasses were sourced from nearby management culls, were confirmed to be disease‐free, and were placed into the field within 24 h of collection. Carcasses weighed an average of 27.64 kg ±1.23 (SE). Within each season, carcasses were spaced at least 1 km apart to minimize scent travel between carcass sites and to maintain a level of spatial independence (Turner et al. 2017). Carcasses were also positioned a minimum of 100 m from any carcass site used in the previous season to reduce the influence of residual olfactory cues and habituation of scavengers to predictable carrion sources (Inagaki et al. 2020; Spencer and Newsome 2021). Carcass placement sites were not strictly randomized; instead, sites were selected using ArcGIS to ensure they were distributed evenly across the matrix of grassland and open forest habitats that comprise the study area, positioned a minimum of 300 m from roads to minimize disturbance, and with sufficient accessibility for placement and monitoring.

Reconyx PC800 Hyperfire battery powered, red flash camera traps (Professional Reconyx Inc., Holmen, WI, USA) were used for road and carcass monitoring. For the road monitoring, cameras were attached to a free‐standing star picket 1–2 m away from the road, at 140 cm height, angled downwards towards the road. For carcass monitoring, cameras were attached to star pickets 3–4 m away from each carcass, at chest height, angled towards the carcass to detect visiting scavengers. To prevent the complete carcass removal from the monitoring frame, each carcass was secured to the ground by tying the neck and Achilles tendon of the animal to two metal stakes spaced 0.6 m apart. Foliage that was directly in front of the road cameras was removed or trimmed away to avoid unnecessary triggers. The cameras were calibrated to take photographs continuously (approximately one image per second) when triggered by thermal movement (i.e., rapid‐fire, no wait period). Individual animals were not identified in this study; thus, detections were analysed at the species level only.

In our study region, dingo home ranges typically span approximately 20–40 km^2^, consistent with estimates from similarly productive habitats in southeastern Australia (Claridge et al. 2009). In contrast, red foxes in comparable regions typically maintain smaller home ranges of approximately 2–10 km^2^ (Meek and Saunders 2000; Carter et al. 2012). Given our 50 km^2^ study area and the placement of 20 carcasses spaced at least 1 km apart, carcasses likely fell within the home ranges of multiple dingoes and foxes, potentially leading to frequent spatial overlap among individuals.

### 2.4 Data Collection

Road camera images were stored, classified to species level where possible, and exported for analysis using the online platform Wildlife Insights (Ahumada et al. 2019). Although the platform uses an artificial intelligence model for image classification, all images were manually reviewed for accuracy. Carcass camera images were manually tagged to species level using Digikam (v7.10.0), and metadata were extracted using the “*camtrap*” package (Niedballa et al. 2020) in the R Statistical Environment (v4.2.0; R Core Development Team 2021). All statistical analyses were also performed in R.

To avoid overestimating activity due to repeated camera triggers by the same individual, images were grouped into independent detection “events.” For road camera images, an event was defined as all dingo or red fox detections that occurred within 1 min of the previous detection of the same species. Detections separated by more than 1 min were classified as new events. This threshold was determined using a histogram of time intervals between consecutive detections, which showed a strong clustering at 1 min, followed by a clear drop off (see Figures S1–S4). For carcass camera images, an event was defined as all dingo or red fox detections that occurred within 10 min of the previous detection of the same species, with detections separated by more than 10 min classified as new events (as per Newsome and Spencer 2022). Carcass sites were monitored continuously for 30 days, a duration known to capture peak scavenger activity in Australian systems (Bragato et al. 2022; Newsome et al. 2024; Newsome and Spencer 2022; Vandersteen et al. 2023). Similarly, in the bioregion this study was undertaken, this period also captures when most of the carcass breakdown process occurs (Spencer and Newsome 2021). All detection events were included in the analyses regardless of whether active scavenging was observed, as our aim was to quantify general activity levels around carrion (as per Cairncross et al. 2024).

### 2.5 Statistical Analysis

#### 2.5.1 Prediction i: Increased Road Activity Following Carcass Deployment

To determine whether dingo and red fox road activity increased after carcass deployment, we modeled the count of independent detection events for each species at road camera sites before and after carcass deployment using generalized linear models (GLMs) with a Poisson distribution. The models included all events recorded within 30 days before and after carcass deployment, with the 30‐day window selected to minimize potential confounding seasonal effects. Two separate GLMs—one for dingo and another one for red fox—were constructed, with the number of detection events as the response variable and with the deployment phase (before or after carcass deployment) and the interaction between the deployment phase and the seasonal monitoring period (winter or summer season) used as the predictor variables. Analyses of variance were then performed on both GLMs to evaluate the overall effect of the variables of interest. GLMs were fitted using the “*glmmTMB*” package (Brooks et al. 2017) and were validated using the “*performance*” package (Lüdecke et al. 2021). An effect was considered significant where *α* < 0.05.

#### 2.5.2 Prediction ii: Temporal Partitioning on Roads

To explore temporal partitioning of dingoes and red foxes on roads, we first calculated diel activity overlap and bootstrapped 95% confidence intervals between both species' activity before and after carcass deployment. Analyses were conducted both across all seasons and within each season independently. Generalized additive models (GAMs) were subsequently used to compare the proportion of activity for each species over a 24‐h period before and after carcass deployment across all seasons and then within seasons. GAMs included proportion of activity as the response variable and a tensor smooth of time of day and an interaction between deployment phase and species (dingo and red fox) as predictors. Models were fitted for the combined dataset and then separately for each season (either winter or summer).

#### 2.5.3 Prediction iii: Temporal Partitioning at Carcass Sites

Temporal partitioning of dingoes and red foxes at carcass sites was assessed using similar methods to those described above. Diel activity overlap and bootstrapped 95% confidence intervals between dingo and red fox activity were first calculated across all seasons and then within seasons. Then, GAMs were used to determine how diel activity patterns varied between species at carcass sites. The first GAM included the proportion of activity as a response variable and a tensor smooth of time of day and the interaction between season and species as predictors. The second GAM included a predicting smooth term of time of day and its interaction with species.

## 3 Results

**FIGURE 2:**
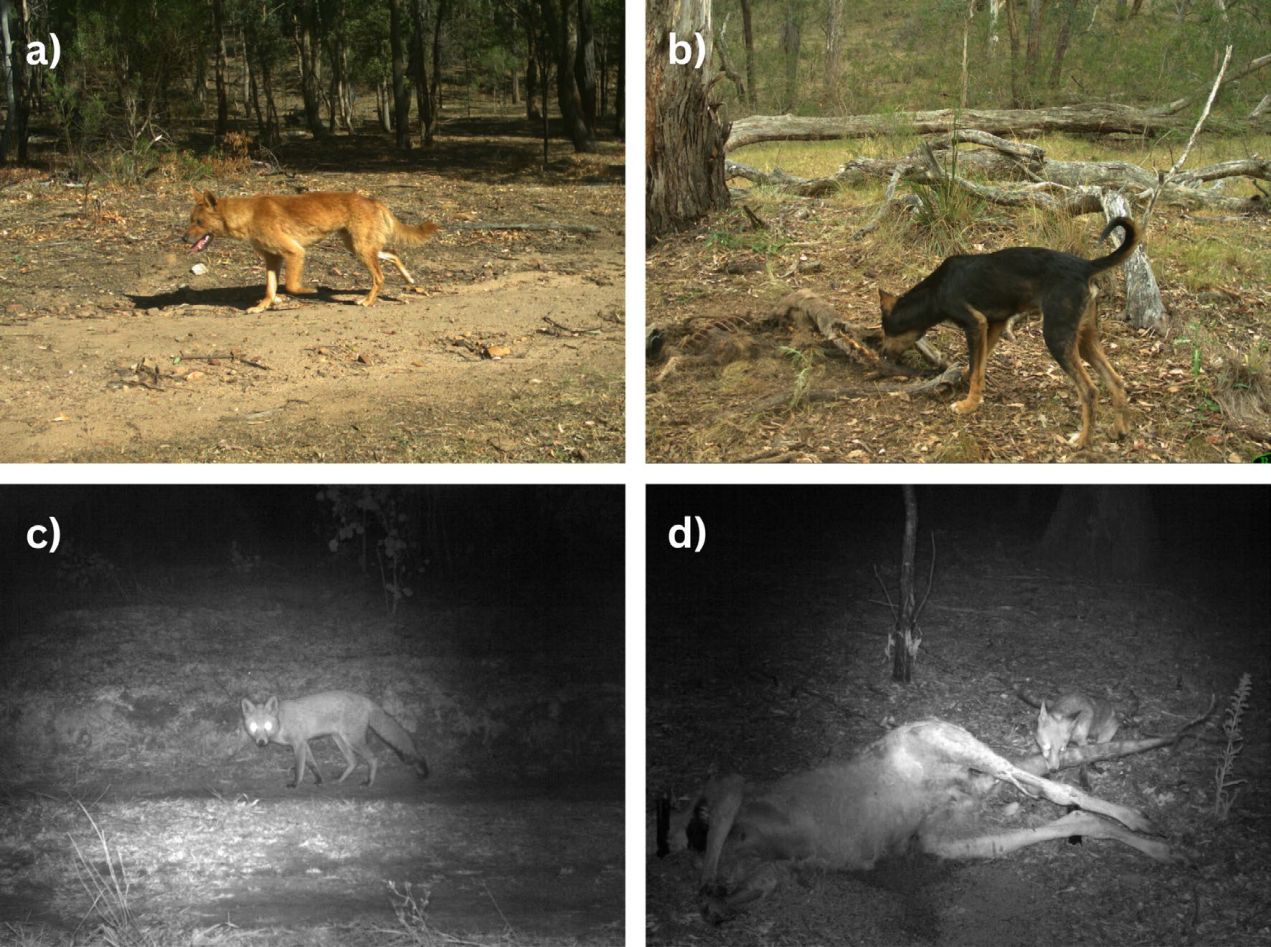
Example camera trap images showing (a) a dingo using a road, (b) a dingo scavenging at a carcass, (c) a red fox using a road, and (d) a red fox scavenging at a carcass.

Across both seasons, 192 dingo and 53 red fox events were recorded on roads (pre‐ and post‐carcass deployment, Figure 2). Red fox road detections were higher in the winter (*n* = 44) than in the summer season (*n* = 9), whereas dingo detections were lower in the winter (*n* = 85) than in the summer season (*n* = 107). At carcass sites, a total of 648 dingo and 220 red fox events were recorded (Figure 2). Detections at carcasses were higher in the winter season for both species (dingoes: winter = 373, summer = 275; red foxes: winter = 208, summer = 12). Full GAM outputs are provided in Tables S2–S5.

### 3.1 Prediction i: Increased Road Activity Following Carcass Deployment

**FIGURE 3:**
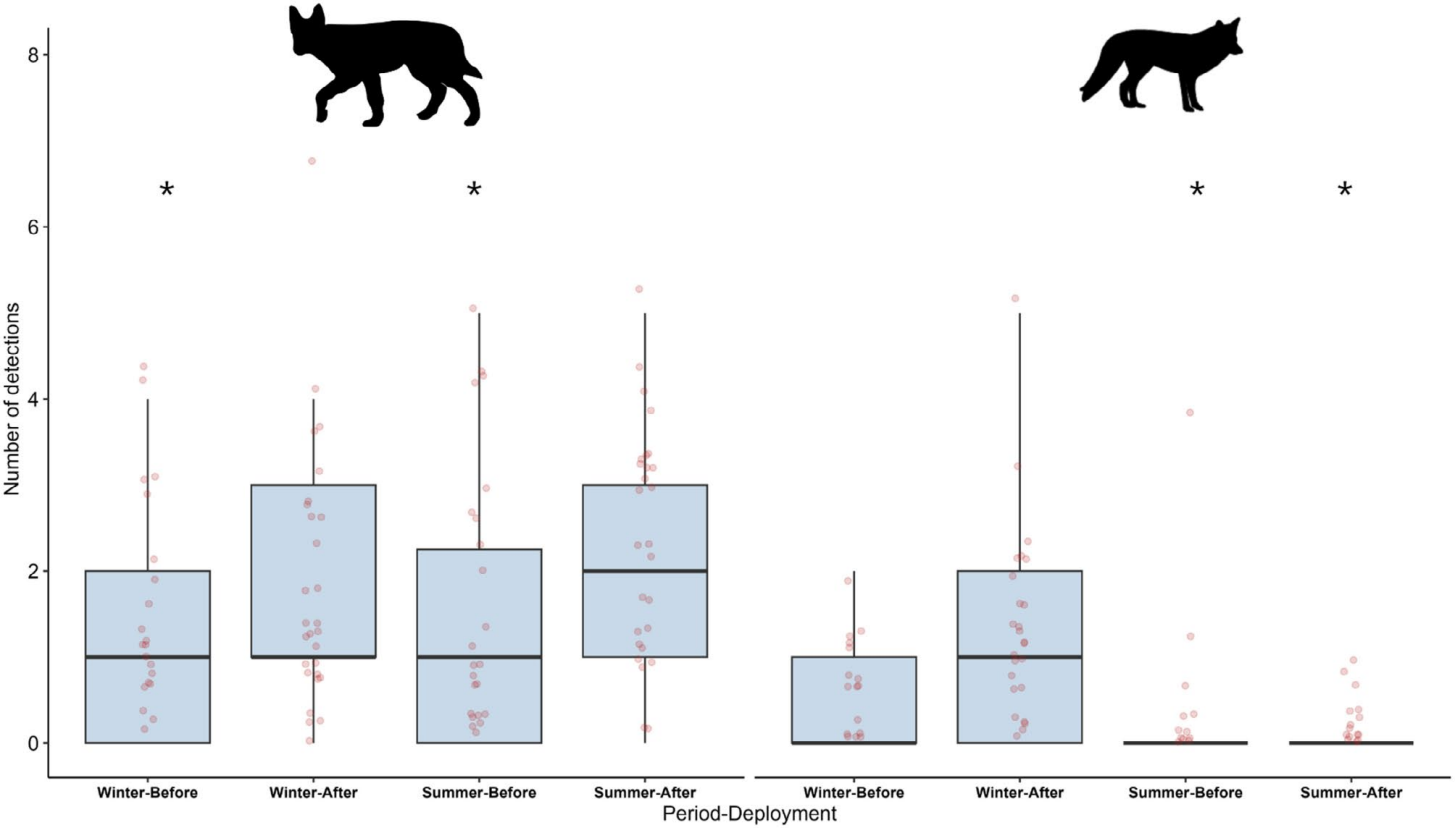
Boxplots showing the number of detection events on road cameras for dingoes and red foxes, before and after carcass deployment, across both seasons. Asterisks indicate variables that were statistically significant. The four boxplots on the left are dingoes and on the right are red foxes.

A significant effect of carcass deployment on dingo road activity was found (*χ* ^2^ = 5.615, *p* = 0.018); but no effect of season (*χ* ^2^ = 2.835, *p* = 0.242). Indeed, dingo road activity increased 0.5‐fold after carcass deployment across both seasons (Table S1; Figure 3).

For red fox road activity, there was a significant interaction between seasons and carcass deployment (*χ* ^2^ = 17.24, *p* < 0.001). Although there was no difference in red fox road detections pre‐carcass deployment (Figure 3, Table S1), red fox detections increased 1.1‐fold across both seasons following carcass deployment (Figure 3, Table S1). This increase in red fox road activity after carcass deployment was 2.3‐fold greater in the winter period compared to the summer period (Figure 3, Table S1).

### 3.2 Prediction ii: Temporal Partitioning on Roads

Overall, temporal overlap between dingoes and red foxes on roads increased post‐carcass deployment (before deployment: Δ = 0.77, LCI = 0.64, UCI = 0.98; after deployment: Δ = 0.87, LCI = 0.81, UCI = 1). In the winter season, however, overlap was lower before carcass deployment (Δ = 0.67, LCI = 0.50, UCI = 0.93) and increased after carcass deployment (Δ = 0.89, LCI = 0.84, UCI = 1). In the summer season, overlap remained low pre‐ and post‐carcass deployment but did increase slightly after carcasses were deployed (before deployment: Δ = 0.19, LCI = 0, UCI = 0.30; after deployment: Δ = 0.28, LCI = 0, UCI = 0.5). All GAM smooths for both pooled and seasonal data showed statistically significant effects (Table S2).

#### 3.2.1 Peak Activity Times—Seasons Combined

**FIGURE 4:**
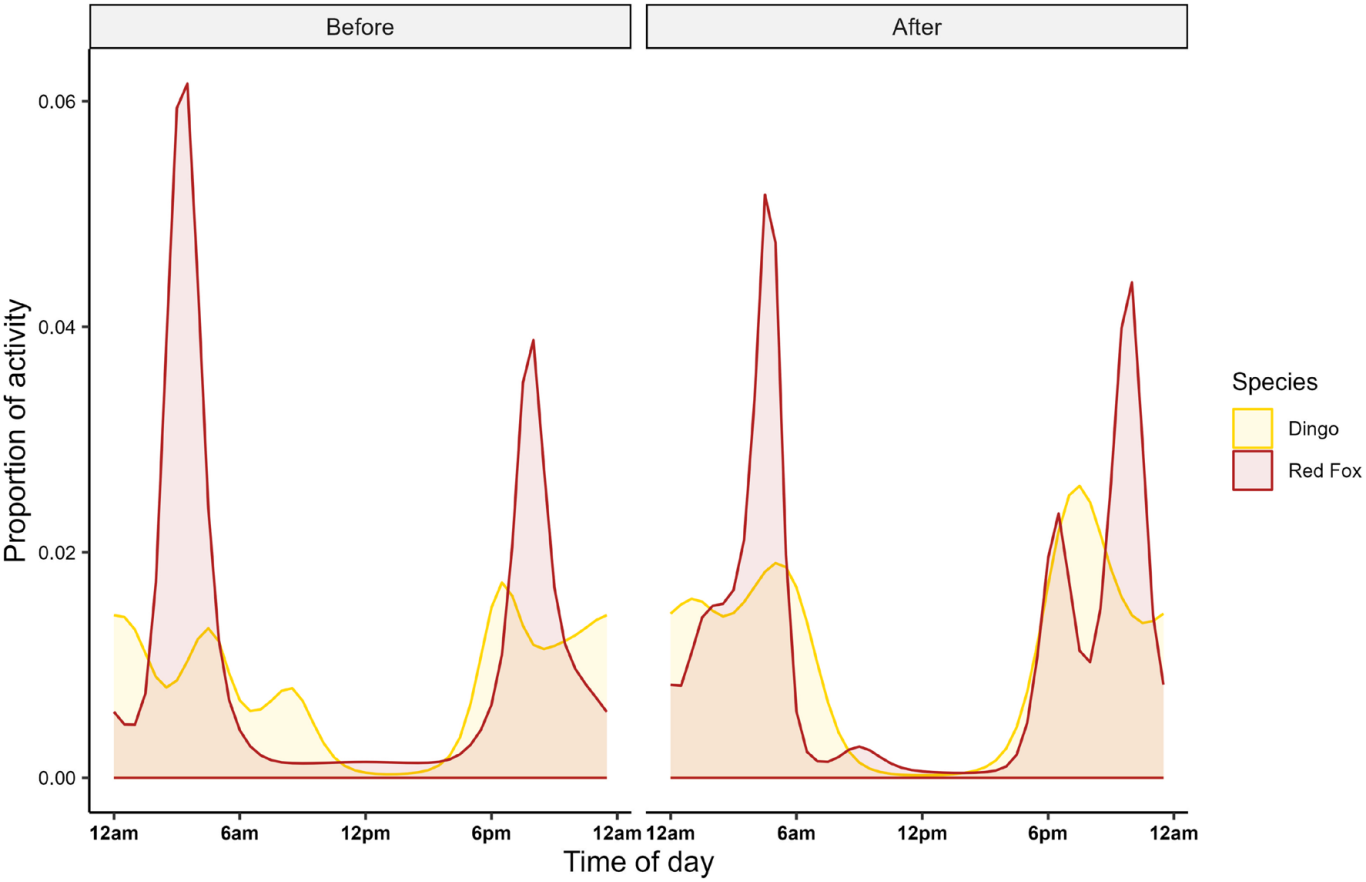
Smooths of proportional activity across the 24‐h period, between dingoes and red foxes, at road monitoring sites, before and after carcass deployment over both seasons.

Before carcass deployment, red foxes exhibited two primary activity peaks on roads: between midnight and dusk (peak activity accounting for approximately 6% of total activity) and shortly after dusk (4% of total activity) (Figure 4). Dingo activity was lower and more variable, with a peak at dusk (1.9% of total activity), midnight (1.6% of total activity), pre‐dawn (1.5% of total activity) and mid‐morning (0.9% of total activity).

After carcass deployment, dingo activity increased with peaks again after dusk (2.9% of total activity), before dawn (1.9% of total activity) and just after midnight (1.7% of total activity). Conversely, red fox activity post‐carcass deployment peaked before dawn, slightly later than prior to the deployment, with the peak accounting for less total activity (5% of total activity). Smaller red fox activity peaks were also observed at dusk (2.4% of total activity) and before midnight (4.7% of total activity). Across seasons, both before and after carcass deployment, dingo and red fox activity peaks remained offset (Figure 4).

#### 3.2.2 Peak Activity Times—Within Season

**FIGURE 5:**
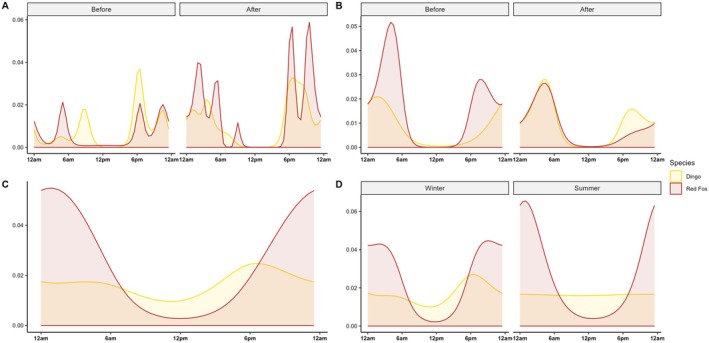
Smooths of proportional activity across the 24‐h period, between dingoes and red foxes, at road monitoring sites, before and after carcass deployment within the (A) winter and (B) summer seasons, and at carcass monitoring sites (C) across both seasons, and (D) within both the winter and summer seasons.

Prior to carcass deployment, dingo and red fox peak activity times on roads overlapped more in the winter (Figure 5A) than in the summer season (Figure 5B). In the summer season, red fox road activity peaked slightly before and after dingo activity. Although in the winter season, dingo activity also peaked diurnally, while red fox activity did not.

Following carcass deployment, differences in peak activity times on roads within seasons for dingoes and foxes were more apparent (Figure 5A,B). In the summer season (Figure 5B), both species' peak activity times overlapped more than in the winter season (Figure 5A), although dingoes, but not red foxes, showed a peak in activity at dusk (Figure 5B). In the winter season (Figure 5A), dingo activity peaked between peaks in red fox activity, between midnight and dawn and after dusk, and red fox activity also peaked once more than dingo activity during the day (Figure 5A).

### 3.3 Prediction iii: Temporal Partitioning at Carcass Sites

Moderate temporal overlap was observed at carcass sites for dingoes and red foxes across seasons (Δ = 0.65, LCI = 0.58, UCI = 0.68). Within seasons, there was lower overlap in the summer (Δ = 0.52, LCI = 0.34, UCI = 0.66) than in the winter season (Δ = 0.64, LCI = 0.56, UCI = 0.68). Modeling proportional activity over a 24‐h period, we found that the smooth terms in the GAM of the combined dataset both exhibited statistical effects (Table S3). But only the smooths that contained red foxes as an interaction term with the different levels of season (winter and summer) also showed a statistical effect when comparing between seasons (Table S3).

#### 3.3.1 Peak Activity Times—Seasons Combined

Overall, red fox activity at carcass sites peaked just after midnight (peak activity accounting for approximately 5.2% of total activity) (Figure 5C). In comparison, dingo carcass activity was much more consistent across the 24‐h period, with one peak evident after dusk (2.6% of total activity). Diurnal activity was also higher during the day for dingoes than for red foxes, with the activity trough in the middle of the day accounting for 1% of total dingo activity compared to 0.3% for red foxes (Figure 5C).

#### 3.3.2 Peak Activity Times—Within Season

During the winter season, red fox activity peaked between dusk and midnight (4.4% of total activity) and again after midnight (4.3% of total activity). Dingo activity at carcass sites peaked at dusk (2.7% of total activity) (Figure 5D).

In the summer season, dingo activity stayed consistent at carcasses across the 24‐h period (~1.7% of total activity occurring at any one time) while red foxes peaked just after midnight (6.3% of activity) (Figure 5D). In both seasons, diurnal dingo activity was higher than diurnal red fox activity (Figure 5D). The relatively unchanging rates of peak activity for dingoes reflected high levels of activity throughout the day and night (*n* = 282 events).

## 4 Discussion

Our study provides new insights into how carcass provisioning influences landscape‐scale road use and activity patterns in two facultative scavengers. Specifically, both dingoes and red foxes increased activity on roads following carcass deployment (Figure 3), supporting *Prediction i* (road use increases post‐carcass deployment). Although this increase was consistent for dingoes across both seasons, red foxes exhibited a stronger response during the winter season, suggesting that they may have greater seasonal dependence on carrion. This dependence is consistent with patterns observed globally, as red foxes have been shown to increase scavenging on ungulate carcasses during cooler months in Scandinavia (Needham et al. 2014) and shift to carrion‐dominant diets in winter in Italy (Cagnacci et al. 2003) and in France (Tobajas et al. 2022). Similar patterns have been recorded for other species, such as in North America where coyotes increase scavenging during winter months (Wilmers et al. 2003).

The rise in road activity following carcass deployment likely demonstrates the behavioral plasticity of scavengers in response to a fluctuating resource. However, it remains unclear whether elevated activity results from increased movements by local dingoes and red foxes or from the attraction of new individuals into the area. Distinguishing between these scenarios could yield insights into local population dynamics and inter and intraspecific competition. Notably, the influx of new individuals could have broader implications for community structure, potentially triggering cascading effects throughout the local ecosystem. Indeed, given the high‐value, nutrient‐dense, and ephemeral nature of carcasses (Baruzzi et al. 2022; Vandersteen et al. 2023; Walker et al. 2021), such resource pulses may act as attractants for scavengers from surrounding areas via olfactory cues, with roads potentially facilitating this increased movement (Finnerty et al. 2022; Naves‐Alegre et al. 2022a).

Carcass provisioning also influenced the temporal activity overlap between scavenging species on roads. In line with *Prediction ii* (reduced temporal separation on roads post‐carcass deployment), dingoes and red foxes exhibited temporal partitioning on roads before carcass deployment, but this separation decreased afterwards, particularly during winter months when their activity times overlapped more (Figure 5A). The increased reliance on carrion by the red fox during winter months, as noted above, may have contributed to this seasonal shift. Carrion, as an unpredictable, ephemeral, and patchily distributed resource, can reduce niche segregation by promoting resource sharing and facilitating coexistence among potential competitors, especially when it is readily available. Such resource pulses may also lead to interspecific information use, where one species benefits from the superior foraging efficiency of another, potentially increasing overlap in their activity patterns (Naves‐Alegre et al. 2022b). However, despite this increased overlap between red foxes and dingoes, their peak activity times remained approximately 1–2 h apart, indicating that temporal partitioning was reduced but not completely eliminated.

These resource‐driven shifts in competitive temporal partitioning parallel findings by Greenville et al. (2014), which showed increased temporal overlap between dingoes and red foxes during a 2‐year resource fluctuation in an Australian desert system. In this study, the top‐down effects of dingoes on red fox activity patterns were more pronounced during “bust” cycles when resources were scarce, leading to greater competitive exclusion of red foxes. Conversely, during “boom” cycles, when resource availability increased, competitive exclusion weakened, and temporal overlap between both species increased. Similar patterns have been observed in North American systems, where resource pulses from elk ( *Cervus canadensis* ) carrion reduce temporal segregation between wolves and coyotes (Wilmers et al. 2003), and in African savannas, where ephemeral carcasses attract multiple predator‐scavenger species, modulating competitive interactions (Hunter et al. 2007). Our findings extend this concept to short‐term resource pulses, showing that even brief carrion influxes can modulate temporal dynamics between dingoes and red foxes.

Although peak activity times at carrion differed between dingoes and red foxes, supporting *Prediction iii* (different peak usage times at carcasses), seasonal variation influenced the extent of temporal overlap between these species (Figure 5C,D). Specifically, as we observed for road activity, overlap in activity at carcass sites was greater during the winter months (Figure 5D). This result may reflect both an increase in red fox reliance on carrion during winter, when alternative food resources become scarce (Needham et al. 2014; Cagnacci et al. 2003; Tobajas et al. 2022), and consistent dingo activity throughout the year. Additionally, across summer months, elevated carcass decomposition rates—driven by higher microbial and invertebrate activity (Payne 1965; Putman 1978)—likely intensified olfactory cues (DeVault and Rhodes 2002; Finnerty et al. 2022), potentially attracting dingoes more frequently during this period, thus suppressing or influencing red fox activity. Furthermore, our summer carcass deployment coincided with the period when dingo pups emerge from dens (approximately October–November) (Catling et al. 1992; Purcell 2010), likely increasing scavenging activity due to greater energetic demands and easy food access for juveniles. In contrast, juvenile red foxes were not observed feeding on carcasses in this study, though the reasons for this remain unclear.

Although road use data has previously been used as an indicator of predator foraging activity (Petridou et al. 2023; Raiter et al. 2018), our results demonstrate that, in this study system, it may also serve as a proxy for facultative scavenging activity linked to carcass provisioning near roads. However, this assumes a spatial association between roads and carrion availability, which may not hold in all ecosystems or landscapes where carcasses are distributed more randomly or further away from roads. In areas where carcasses may commonly occur near roads, future research could focus on identifying individual scavengers detected on roads and carcasses to determine whether increased road use reflects heightened activity of existing individuals, an influx of new animals drawn to the resource, or a combination of both. This distinction can inform both community dynamics and movement ecology but may be less critical for understanding decomposition processes alone. In addition, a better understanding of how background carrion availability and its distribution across the landscape influence scavenger movement and activity patterns would assist in determining whether there is a threshold carcass density that generates landscape‐scale changes in scavenger behavior. In our case, the supplementation of 20 additional kangaroo carcasses (~550 kg of carrion) over an area of 2480 ha, generated an increase in activity for two facultative scavengers. This suggests that even small increases in carrion loads can influence scavenger activity, but varying the carrion loads in future studies would assist this interpretation.

By broadening scavenging research beyond localized carcass sites to include broader‐scale activity patterns within our study area, we take an important step toward understanding the ecological significance of scavenger interactions and the role of carrion in shaping predator–prey dynamics. Moreover, our results underscore the ecological significance of even small resource pulses in shaping competitive interactions between scavenging species. By demonstrating that carrion availability can influence spatial overlap, activity patterns, and competitive dynamics between dingoes and red foxes, our study contributes to a growing body of research emphasizing the role of carrion in structuring ecological communities. Future research should investigate how these patterns may vary across different scavenger guilds, habitats, and anthropogenic landscapes, particularly in the context of expanding road networks and human‐modified food resources.

Moreover, our findings have important conservation implications. Increased scavenger use of roads in response to carrion provisioning may elevate the risk of vehicle collisions, particularly for native apex predators such as dingoes. This risk underscores the need to balance carrion management and road safety considerations, especially in areas where scavenger species are vulnerable or play critical ecological roles. Future management should consider how carrion placement or removal near roads may influence wildlife–vehicle collision rates and broader ecosystem dynamics. Finally, the fact that red foxes showed a landscape‐scale response to carrion availability, and frequently utilized carrion as a food resource (especially in winter), underscores the need to understand the extent to which carrion supports their populations and exacerbates their broader ecological effects as an invasive species in Australia. Future management should therefore also consider whether there is a need to manage carcass loads in areas or at times when red fox impacts are of concern.

## Author Contributions

**Patrick B. Finnerty:** formal analysis (supporting), writing – original draft (lead), writing – review and editing (lead). **Niraj Y. Meisuria:** data curation (equal), formal analysis (supporting), writing – original draft (supporting), writing – review and editing (equal). **Luke J. Baker:** data curation (lead), formal analysis (supporting), investigation (equal), writing – original draft (supporting), writing – review and editing (equal). **Rhys J. Cairncross:** data curation (equal), formal analysis (lead), writing – original draft (supporting), writing – review and editing (equal). **Emma Spencer:** conceptualization (equal), data curation (equal), funding acquisition (equal), investigation (equal), methodology (equal), writing – review and editing (equal). **Mathew S. Crowther:** supervision (equal), writing – review and editing (equal). **Thomas M. Newsome:** conceptualization (equal), funding acquisition (equal), project administration (equal), supervision (equal), writing – review and editing (supporting).

## Conflicts of Interest

The authors declare no conflicts of interest.

## Supporting information


**Table S1:** Output from GLM assessing red fox and dingo detections on roads before and after carcass deployment across seasons. Bold values indicate a statistical effect at α < 0.05.
**Table S2:** Output from the GAMs predicting the proportion of activity for dingoes (49.2% deviance explained) and red foxes (35.2% deviance explained) across the 24‐h period, before and after carcass deployments and across seasonal periods.
**Table S3:** Output from the GAMs predicting the proportion of activity for dingoes (34.2% deviance explained) and red foxes (36.9% deviance explained) across the 24‐h period, at road and carcass sites and across seasonal periods.
**Table S4:** Output from the GAMs predicting the proportion of activity for dingoes and red foxes on roads across all seasons (39.5% deviance explained), the Winter (63.4% deviance explained) and Summer seasonal periods (29% deviance explained) across the 24‐h period, before and after deployment of carcasses.
**Table S5:** Output from the GAMs predicting the proportion of activity for dingoes and red foxes at carrion across all seasons (38.2% deviance explained) and within the Winter and Summer seasonal periods (39.8% deviance explained) across the 24‐h period.
**Figure S1:** Histogram of time difference values between detections of dingoes on roads. The minimum time difference value that accounted for most of the data was chosen to increase resolution of activity levels.
**Figure S2:** Histogram of time difference values between detections of red foxes on roads. The minimum time difference value that accounted for most of the data was chosen to increase resolution of activity levels.
**Figure S3:** Histogram of time difference values between detections of dingoes on carcasses. The minimum time difference value that accounted for most of the data was chosen to increase resolution of activity levels.
**Figure S4:** Histogram of time difference values between detections of red foxes on carcasses. The minimum time difference value that accounted for most of the data was chosen to increase resolution of activity levels.
**Data S1:** ece372059‐sup‐0001‐supinfo.zip.
**Data S2:** ece372059‐sup‐0001‐supinfo.zip.

## Data Availability

All data, metadata, and code have been uploaded as Supporting Information.
