# Supplementary material for: Carrion Increases Landscape‐Scale Scavenger Activity and Interactions
Source: Ecol Evol. 2025 Aug 23;15(8):e72059. doi: 10.1002/ece3.72059 (PMC12374251; doi:10.1002/ece3.72059)
Supplement: Supplementary file 1 — Table S1: Output from GLM assessing red fox and dingo detections on roads before and after carcass deployment across seasons. Bold values indicate a statistical effect at α < 0.05. Table S2: Output from the GAMs predicting the proportion of activity for dingoes (49.2% deviance explained) and red foxes (35.2% deviance explained) across the 24‐h period, before and after carcass deployments and across seasonal periods. Table S3: Output from the GAMs predicting the proportion of activity for dingoes (34.2% deviance explained) and red foxes (36.9% deviance explained) across the 24‐h period, at road and carcass sites and across seasonal periods. Table S4: Output from the GAMs predicting the proportion of activity for dingoes and red foxes on roads across all seasons (39.5% deviance explained), the Winter (63.4% deviance explained) and Summer seasonal periods (29% deviance explained) across the 24‐h period, before and after deployment of carcasses. Table S5: Output from the GAMs predicting the proportion of activity for dingoes and red foxes at carrion across all seasons (38.2% deviance explained) and within the Winter and Summer seasonal periods (39.8% deviance explained) across the 24‐h period. Figure S1: Histogram of time difference values between detections of dingoes on roads. The minimum time difference value that accounted for most of the data was chosen to increase resolution of activity levels. Figure S2: Histogram of time difference values between detections of red foxes on roads. The minimum time difference value that accounted for most of the data was chosen to increase resolution of activity levels. Figure S3: Histogram of time difference values between detections of dingoes on carcasses. The minimum time difference value that accounted for most of the data was chosen to increase resolution of activity levels. Figure S4: Histogram of time difference values between detections of red foxes on carcasses. The minimum time difference value that accounted for most o [file ECE3-15-e72059-s001.zip › ece372059-sup-0001-supinfo.docx]

**Supplementary material**

Table S1: Output from GLM assessing red fox and dingo detections on roads before and after carcass deployment across seasons. Bold values indicate a statistical effect at α < 0.05.

| Variable | Estimate | SE | Z-value | p-value |
| --- | --- | --- | --- | --- |
| Red fox | | | | |
| Intercept | 0.129 | 0.174 | 0.742 | 0.458 |
| Deployment-Before | -1.099 | 0.348 | -3.156 | **0.002*** |
| Deployment-After * Period-Summer | -2.398 | 0.603 | -3.976 | **<0.001*** |
| Deployment-Before * Period-Summer | -0.606 | 0.508 | -1.194 | 0.232 |
| Dingo | | | | |
| Intercept | 0.584 | 0.139 | 4.211 | **< 0.001*** |
| Deployment-Before | -0.455 | 0.223 | -2.043 | **0.041*** |
| Deployment-After * Period-Summer | 0.160 | 0.189 | 0.846 | 0.398 |
| Deployment-Before * Period-Summer | 0.332 | 0.228 | 1.456 | 0.145 |

Table S2: Output from the GAMs predicting the proportion of activity for dingoes (49.2% deviance explained) and red foxes (35.2% deviance explained) across the 24-hour period, before and after carcass deployments and across seasonal periods.

| Variable | e.d.f | Reference d.f. | F-value | p-value |
| --- | --- | --- | --- | --- |
| Dingo | | | | |
| Deployment-After * Period-Winter | 4.991 | 8 | 6.060 | **< 0.001*** |
| Deployment-Before * Period-Winter | 6.414 | 8 | 3.850 | **< 0.001*** |
| Deployment-After * Period-Summer | 7.948 | 8 | 6.210 | **< 0.001*** |
| Deployment-Before * Period-Summer | 2.805 | 8 | 3.820 | **< 0.001*** |
| Red fox | | | | |
| Deployment-After * Period-Winter | 3.000 | 3 | 8.900 | **< 0.001*** |
| Deployment-Before * Period-Winter | 2.876 | 3 | 3.604 | **0.012*** |
| Deployment-After * Period-Summer | 2.926 | 3 | 7.181 | **< 0.001*** |
| Deployment-Before * Period-Summer | 3. 000 | 3 | 10.324 | **< 0.001*** |

Table S3: Output from the GAMs predicting the proportion of activity for dingoes (34.2% deviance explained) and red foxes (36.9% deviance explained) across the 24-hour period, at road and carcass sites and across seasonal periods.

| Variable | e.d.f | Reference d.f. | F-value | p-value |
| --- | --- | --- | --- | --- |
| Dingo | | | | |
| Location-Carcass * Period-Winter | 2.600 | 3 | 2.498 | **0.039*** |
| Location-Road * Period-Winter | 2.945 | 3 | 16.115 | **< 0.001*** |
| Location-Carcass * Period-Summer | 0.485 | 3 | 0.220 | 0.2558 |
| Location-Road * Period-Summer | 2.920 | 3 | 13.064 | **< 0.001*** |
| Red fox | | | | |
| Location-Carcass * Period-Winter | 2.993 | 3 | 8.865 | **< 0.001*** |
| Location-Road * Period-Winter | 2.994 | 3 | 8.656 | **< 0.001*** |
| Location-Carcass * Period-Summer | 2.631 | 3 | 12.156 | **< 0.001*** |
| Location-Road * Period-Summer | 2.997 | 3 | 14.201 | **< 0.001*** |

Table S4: Output from the GAMs predicting the proportion of activity for dingoes and red foxes on roads across all seasons (39.5% deviance explained), the Winter (63.4% deviance explained) and Summer seasonal periods (29% deviance explained) across the 24-hour period, before and after deployment of carcasses.

| Variable | e.d.f | Reference d.f. | F-value | p-value |
| --- | --- | --- | --- | --- |
| All seasons | | | | |
| Deployment-After * Species-Dingo | 6.034 | 18 | 1.820 | **< 0.001*** |
| Deployment-Before * Species-Dingo | 7.996 | 18 | 0.934 | **0.022*** |
| Deployment-After * Species-Red fox | 11.085 | 18 | 3.610 | **< 0.001*** |
| Deployment-Before * Species-Red fox | 9.104 | 18 | 4.150 | **< 0.001*** |
| Winter season | | | | |
| Deployment-After * Species-Dingo | 9.379 | 18 | 2.585 | **< 0.001*** |
| Deployment-Before * Species-Dingo | 9.509 | 18 | 2.108 | **< 0.001*** |
| Deployment-After * Species-Red fox | 14.401 | 18 | 4.479 | **< 0.001*** |
| Deployment-Before * Species-Red fox | 9.614 | 18 | 1.477 | **0.002*** |
| Summer season | | | | |
| Deployment-After * Species-Dingo | 2.980 | 3 | 4.017 | **< 0.001*** |
| Deployment-Before * Species-Dingo | 2.184 | 3 | 3.464 | **< 0.001*** |
| Deployment-After * Species-Red fox | 2.736 | 3 | 3.830 | **< 0.001*** |
| Deployment-Before * Species-Red fox | 3.000 | 3 | 8.858 | **< 0.001*** |

Table S5: Output from the GAMs predicting the proportion of activity for dingoes and red foxes at carrion across all seasons (38.2% deviance explained) and within the Winter and Summer seasonal periods (39.8% deviance explained) across the 24-hour period.

| Variable | e.d.f | Reference d.f. | F-value | p-value |
| --- | --- | --- | --- | --- |
| All seasons | | | | |
| Species-Dingo | 2.661 | 3 | 2.593 | **0.036*** |
| Species-Red fox | 2.808 | 3 | 31.560 | **< 0.001*** |
| Winter and Summer season | | | | |
| Species-Dingo * Period-Winter | 2.472 | 3 | 1.787 | 0.095 |
| Species-Red fox * Period-Winter | 2.901 | 3 | 15.095 | **< 0.001*** |
| Species-Dingo * Period-Summer | 0.144 | 3 | 0.051 | 0.348 |
| Species-Red fox * Period-Summer | 2.225 | 3 | 24.084 | **< 0.001*** |


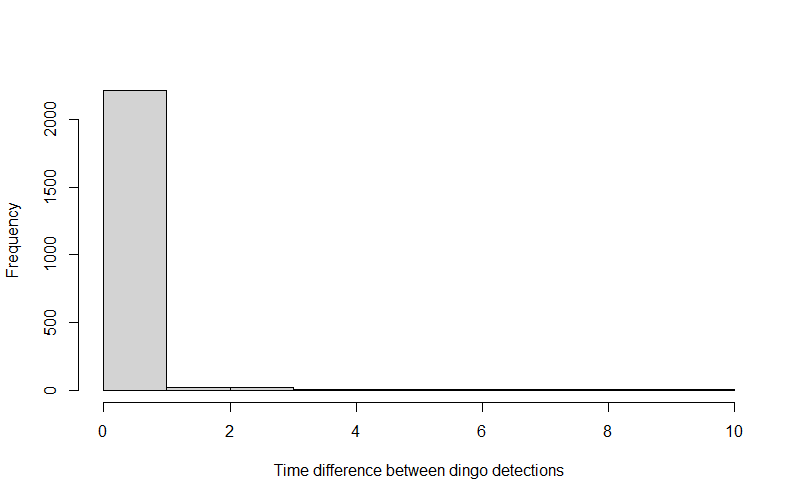


Figure S1: Histogram of time difference values between detections of dingoes on roads. The minimum time difference value that accounted for most of the data was chosen to increase resolution of activity levels.


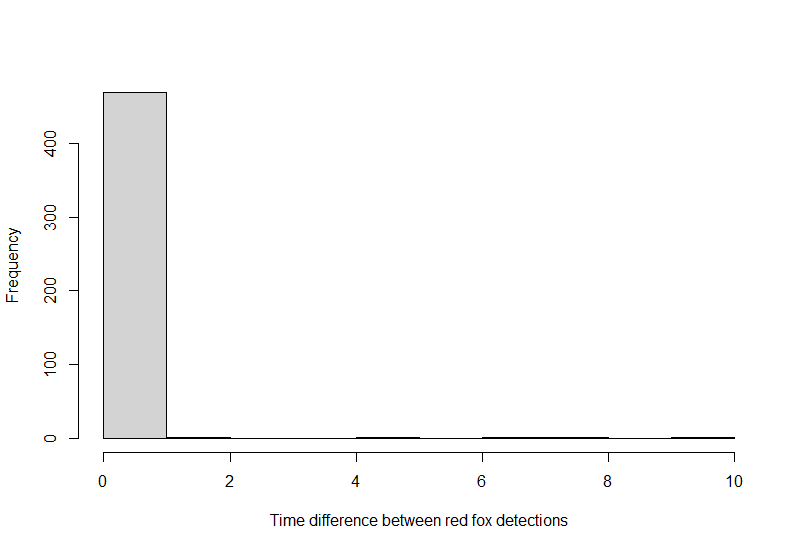


Figure S2: Histogram of time difference values between detections of red foxes on roads. The minimum time difference value that accounted for most of the data was chosen to increase resolution of activity levels.


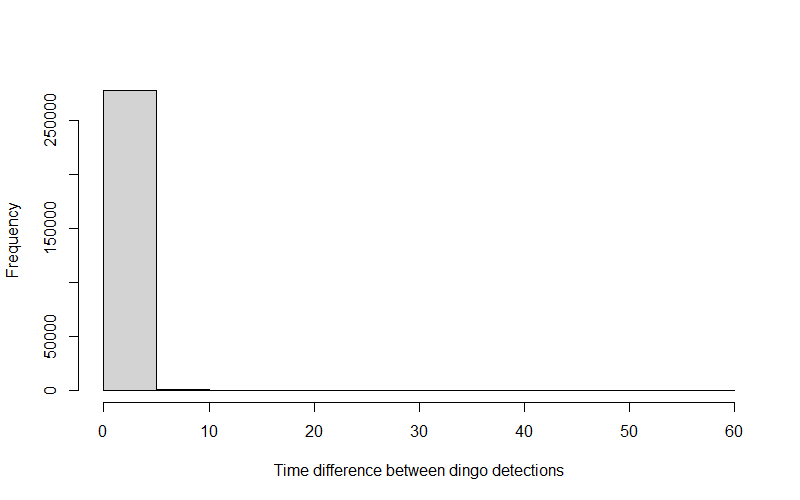


Figure S3: Histogram of time difference values between detections of dingoes on carcasses. The minimum time difference value that accounted for most of the data was chosen to increase resolution of activity levels.


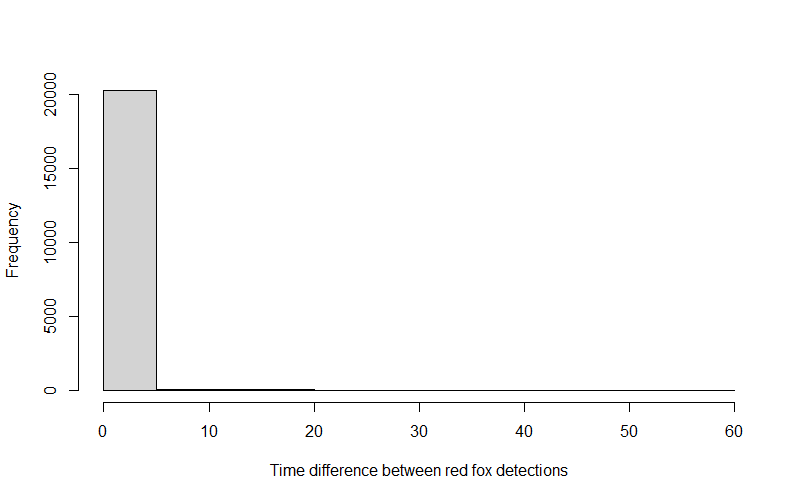


Figure S4: Histogram of time difference values between detections of red foxes on carcasses. The minimum time difference value that accounted for most of the data was chosen to increase resolution of activity levels.
